# A comparative study between two antifungal agents, Luliconazole and Efinaconazole, of their preventive effects in a *Trichophyton*-infected guinea pig onychomycosis model

**DOI:** 10.1093/mmy/myaa111

**Published:** 2021-02-04

**Authors:** Akihiro Nakamura, Satoko Hirakawa, Hiroaki Nagai, Katsuhiro Inagaki

**Affiliations:** Research Center, Nihon Nohyaku Co., Ltd., Kawachi-Nagano, Osaka, Japan; Research Center, Nihon Nohyaku Co., Ltd., Kawachi-Nagano, Osaka, Japan; Research Center, Nihon Nohyaku Co., Ltd., Kawachi-Nagano, Osaka, Japan; Research Center, Nihon Nohyaku Co., Ltd., Kawachi-Nagano, Osaka, Japan

**Keywords:** luliconazole, efinaconazole, *Trichophyton mentagrophytes*, guinea pig, lasting antifungal effect

## Abstract

An efficacious period of two topical antifungal drugs was compared in a *Trichophyton mentagrophytes*-infected onychomycosis model in guinea pigs treated with antifungal drugs prior to infection. Luliconazole 5% (LLCZ) and efinaconazole 10% (EFCZ) test solutions were applied to the animals’ nails once daily for 2 weeks followed by a nontreatment period of 2, 4, and 8 weeks. After each nontreatment period, the nails were artificially infected by the fungus. Drug efficacy was quantitatively evaluated by qPCR and histopathological examination of the nails collected following a 4-week post-infection period. The fungal infection was confirmed in the untreated group. Both LLCZ and EFCZ prevented fungal infection in the treated groups with the nontreatment period of 2 weeks. After the nontreatment period of 4 weeks, no infection was observed in the LLCZ-treated group; however, infection into the nail surface and fungal invasion into the nail bed were observed in the EFCZ-treated group. After the nontreatment period of 8 weeks, fungi were found in the nail surface and nail bed in some nails treated with EFCZ; however, no infection was observed in the nail bed of the LLCZ-treated group. The results suggest that LLCZ possesses longer-lasting antifungal effect in nails of the guinea pigs than EFCZ, and that this animal model could be useful for translational research between preclinical and clinical studies to evaluate the pharmacological efficacy of antifungal drugs to treat onychomycosis. This experimentally shown longer-lasting preventive effects of LLCZ could also decrease the likelihoods of onychomycosis recurrence clinically.

## Introduction

Onychomycosis is a common nail disorder and the most serious infections by fungi, *Trichophyton rubrum* and *Trichophyton interdigitale,* in the nail plate and nail bed. The prevalence rates are around 10–16% with affected populations in all ages.^1–4^ In the literature, a recurrence (relapse [the same infection occurring after incomplete cure] / reinfection [the same infection after complete cure]) rate of 20%–25% has been noted following initial successful treatment of onychomycosis with systemic or topical antifungal drugs.^5–7^

Many authors have pointed out that a prophylactic regimen of topical antifungal drugs would be effective to prevent the recurrence.^5^^,^^8–12^ However, there have not been many marketed topical antifungal drugs to confirm this medical concept. Efinaconazole and luliconazole are newly launched topical drugs for the treatment of onychomycosis in Japan and/or United States, and these drugs can be used to confirm the prophylactic concept.

Efinaconazole and luliconazole are both classified as azole-structured compounds. Efinaconazole 10% solution (EFCZ) for a topical treatment of onychomycosis is approved in the United States, Canada, Korea, and Japan. On the other hand, luliconazole 5% solution (LLCZ) for a topical onychomycosis treatment is approved in Japan. This compound has a high *in vitro* antifungal activity against *Trichophyton spp*.^13^^,^^14^ In addition, these two compounds are kinetically different in their affinity for keratin, their permeability into the deeper tissue from the applied nail surface, and their ability to remain within the tissue.^11^^,^^15^^,^^16^ In this study, we evaluated these two topical antifungal drugs for their preventive effects from fungal infection into the nail tissue and their effective period after their nail treatment in a guinea pig onychomycosis model.

Guinea pig and rabbit models have been used to evaluate the efficacy of antifungal agents that have been reported in the cure of onychomycosis.^15^^,^^17–21^ Tatsumi et al reported evaluations of some antifungal agents using a guinea pig model of onychomycosis.^17^ Although this paper was the first paper reporting an animal model used for the curative efficacy of onychomycosis, detailed distributions of fungus elements in the nail tissue were not clearly described with a histopathological approach. Shimamura et al. reported that a novel model of onychomycosis in rabbit showed some disease features commonly shared within many onychomycosis patients.^18^ Their consistent findings in the model were similar to the clinical diagnoses of proximal subungual type (PSO), the distal subungual type (DSO) and the superficial white onychomycosis (SWO). In this study, we did not only show the *Trichophyton mentagrophytes*-infected guinea pig onychomycosis model with the features which had been clinically seen in onychomycosis patients, but also evaluated the drug efficacy quantitatively by fungal DNA and fungal distributions in the nail tissue by staining fungi and evaluating histopathologically. This disease model that was established with small animals (guinea pig) and with those two different evaluation methods using quantitative polymerase chain reaction (qPCR) and histopathology offer sufficient but rather a quick approach to conduct preclinical experiments and yield sufficiently quantified results to compare these two newly launched antifungal drugs in their preventive effects against fungal infection.

## Methods

### Animal model

A total of 27 male Hartley strain Specific Pathogen-Free guinea pigs (Japan SLC, Shizuoka, Japan) aged 5 weeks old were used. The guinea pigs were housed individually in wire mesh cages throughout the experimental period in a room temperature under a 12:12-h of light:dark cycle. They were given access to a pelletized diet and tap water *ad libitum*. The animals were housed in a negative pressured rack (CLEA Japan, Tokyo, Japan) during fungal infection. The animal experiment was approved by the animal welfare committee at Research Center, Nihon Nohyaku Co., Ltd. The institute is accredited with the Center for Accreditation of Laboratory Animal Care and Use, Japan Health Sciences Foundation that promotes the humane treatment of animals in sciences (Tokyo, Japan). Animal care and use conformed to the principles established by the animal welfare committee of the institute and complied with all legal requirements for the humane treatment and management of the animals.

### Fungal Isolates

*T. mentagrophytes* TIMM2789 strain was obtained from the Teikyo University Institute of Medical Mycology, Tokyo, Japan. Sabouraud dextrose agar (SDA; Becton Dickinson, Maryland, the United States) was used for the preculturing of the fungal species. For inoculum preparation, after the fungal isolates were grown on agar slant at a temperature of 27°C for a period of 2 weeks, sterile normal saline with 0.1% (v/v) Tween80 was added to the slants, and isolate's conidia was suspended by rubbing the colony gently with a loop. The suspension was filtered through a sterile gauze to remove hyphal fragments. Most of the inoculum were microconidia, however, the inoculum contained a small number of macroconidia but no arthroconidia. The number of conidia in the filtrate was counted using a Thoma hemacytometer, and its concentration was adjusted to 1.0 × 10^8^ conidia/mL using Sabouraud Dextrose Broth (SDB; Becton Dickinson, Maryland, the United States).

### Drug samples

Luliconazole 5% solution (LLCZ, Luconac^®^, Topical Solution 5%) and efinaconazole 10% solution (EFCZ, Clenafin^®^, Topical Solution 10%) were commercially purchased. Triamcinolone Acetonide was used for immune suppression during the inoculation period.

### Production of a *T. mentagrophytes*-infected guinea pig onychomycosis model

As the scientific literature about the natural onychomycosis in the animal species has not been present, a *T. mentagrophytes*-infected guinea pig onychomycosis model was produced.

**Figure 1.**
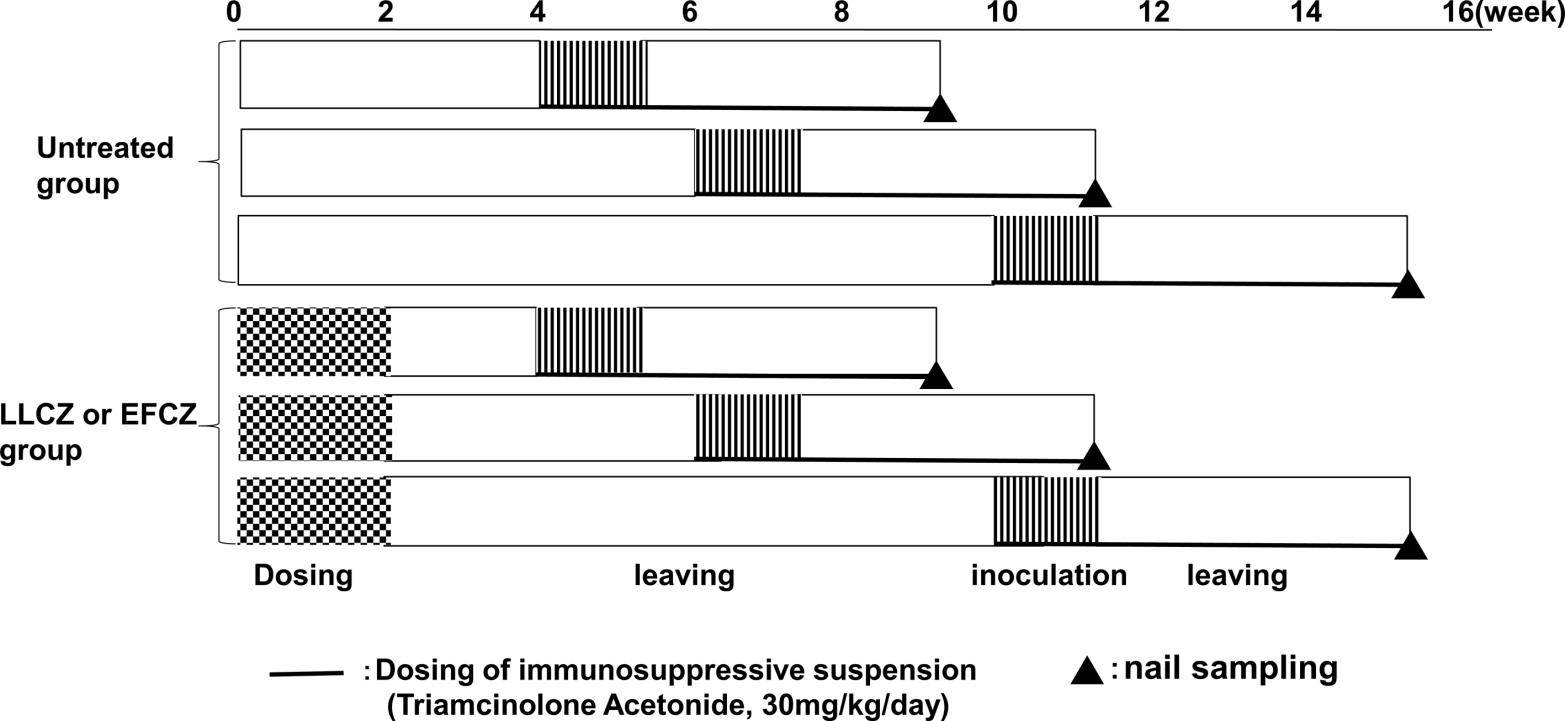
Schematic time schedule of the animal experiment. LLCZ, Luliconazole 5% solution; EFCZ, Efinaconazole 10% solution.

The animals were assigned to three groups (untreated, LLCZ, and EFCZ) composed of nine animals each (Fig. 1). For the LLCZ and EFCG groups, all three nails (from the second, third, and fourth toes) per hindfoot were individually treated with 5 µl of either drug (LLCZ or EFCZ) once daily for 2 weeks. After the completion of the drug treatments, animals were divided into further sub-groups to be left untreated for 2, 4, and 8 weeks followed by fungal inoculation (Fig. 1). Following the nontreatment period, the nails were inoculated with the fungal microconidia (2 × 10^7^ conidia/foot). The mesh of a bandage (Band-AID^®^, Johnson & Johnson, Tokyo, Japan) soaked with 0.2 mL of conidia suspension was used to cover the second, third, and fourth toes. The toes were further covered with a plastic wrap, a foam pad using an adhesive compound (1 by 2 cm, 1560 M, 3 M Japan Health Care, Tokyo, Japan) was put on the heel, and then, fixed with an adhesive elastic tape (Tensoplast^®^; BSN medical GmbH, Pinetown, Germany). Finally, to reinforce this condition, the toes were wrapped over by surgical tapes (No.12, Nichiban Tokyo, Japan).

This condition was maintained for 1 week as the inoculation phase. All materials used for the inoculation were removed after the phase, and the nontreatment state was maintained for 4 weeks as the postinfection period. Triamcinolone acetonide was intramuscularly administrated at a dose of 30 mg/kg once daily for 5 weeks from the day of fungal inoculation to the day of the post-infection period. After the post-infection period was completed, the animals were sacrificed by intraperitoneal injection of pentobarbital, and the nails were removed from their toes. The removed nail samples were supplied for qPCR analysis and histopathological examination.

### Quantitative real-time polymerase chain reaction assay

Quantitative real-time PCR (qPCR) assay using the internal transcribed spacer (ITS) region of *T. mentagrophytes* DNA was used to detect the viability of the fungi.^22^ qPCR has comparable sensitivity to potassium hydroxide (KOH) examination and nail culture assay by colony-forming unit.^23–25^ A total of 12 nails per group (1 or 2 nails per foot) was measured.

The preparation of a standard curve of qPCR, and nucleic acid extraction and quantification from conidia were performed by the method reported previously^24^ with slight modifications. DNA extraction from the nails was performed using ISOHAIR protocol (Nippon Gene, Tokyo, Japan). DNA extracted from one whole nail was dissolved in 40 µl of Tris-EDTA buffer. As qPCR was performed with 1 µl of this obtained DNA solution, the number of conidia DNA copy per nail was calculated by multiplying obtained data with 40.

In addition, we determined that the fixed-quantity minimum limit value of *T. mentagrophytes* per nail is 10,000 copies of ITS DNA because the primers for the ITS region of *T. mentagrophytes* DNA form the primer-dimer below 10,000 copies.^22^

### Histopathological examination of nail tissue

The nails (one nail per foot) sampled for histopathological examination were fixed in 10% (*v/v*) buffered neutral formalin solution and embedded in paraffin wax. The nails were serially sectioned at 5 µm in thickness using a microtome to support the cohesion of the whole-nail elements by Kawamoto's film method.^26^ The cut thin paraffin sections were stained with the Periodic acid-Schiff (PAS) stain or Fungiflora Y kit stain^27^ (Trustmedical, Hyogo, Japan) and were observed by light or fluorescence microscopy. Briefly, Fungiflora Y stain method was described as follows. The blocking buffer was dropped and spread to nail pieces. The staining buffer was dropped and spread to nail pieces following washing after a few minutes. The pieces were transferred to alcoholic dehydration following washing. All nails for histopathological examination were evaluated whether fungi exist, and the infection rates were calculated.

### Statistical analysis

The log_10_ of the number of copies of ITS DNA in the nails by qPCR were analyzed by the Steel-Dwass test. On the other hand, the infection rate was calculated by dividing the number of nails in which fungi was found in the histopathological examination by the number of nails examined. The infection rate in each group was analysed using the one-way Fisher's exact test (one-sided test). *P* values of less than 0.05 were regarded as significant.

## Results

### Quantification of *T. mentagrophytes* by qPCR

**Figure 2.**
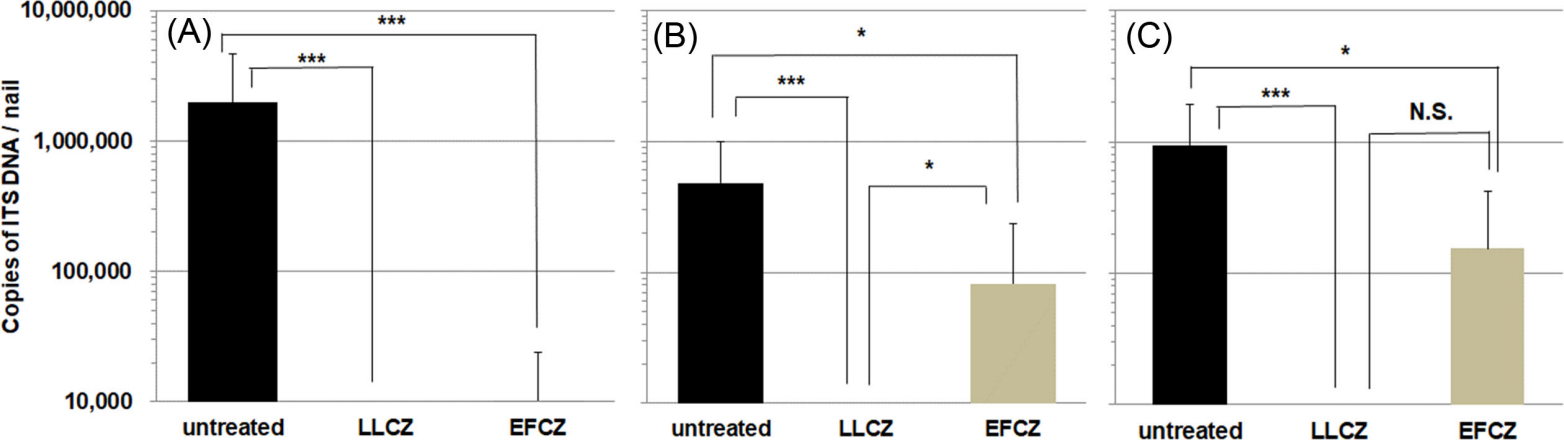
Differences in the number of copies of ITS DNA in each subgroup using qPCR. The number of copies of ITS DNA in the nails untreated (N = 12) and treated with antifungal drugs (N = 12 for LLCZ and EFCZ). Groups with 2 weeks (A), 4 weeks (B), and 8 weeks (C) of the non-treatment period after dosing with respective antifungal drug to nails of the guinea pig hind feet. The log_10_ of the number ITS region copies by qPCR in nails were analyzed using the Steel-Dwass test. *P* values of less than .05 were regarded as significant. Mean copies of ITS DNA are shown along with the associated standard deviation values. ^*^P < .05, ^***^P < .001, ITS; internal transcribed spacer, N.S.; not significant. LLCZ, Luliconazole 5% solution; EFCZ, Efinaconazole 10% solution.

The number of copies of ITS DNA in the nails treated with LLCZ and EFCZ was significantly lower than that of the untreated in all subgroups with the nontreatment period of 2, 4, or 8 weeks (Fig. 2). In the subgroups with 4 weeks of non-treatment period, the number of copies of ITS DNA in the nails treated with EFCZ was significantly higher than that of the group of LLCZ (Fig. 2B). This difference between the two treated groups was also observed in subgroups with 8 weeks of nontreatment period; however, there was no statistically significant difference between the treated groups (*P* = .11, Fig. 2C). The mean value of the number of copies of ITS DNA in the nails treated with LLCZ was lower than the fixed-quantity limit value in all subgroups.

### Histopathological characterization and infection rate of the untreated group

**Figure 3.**
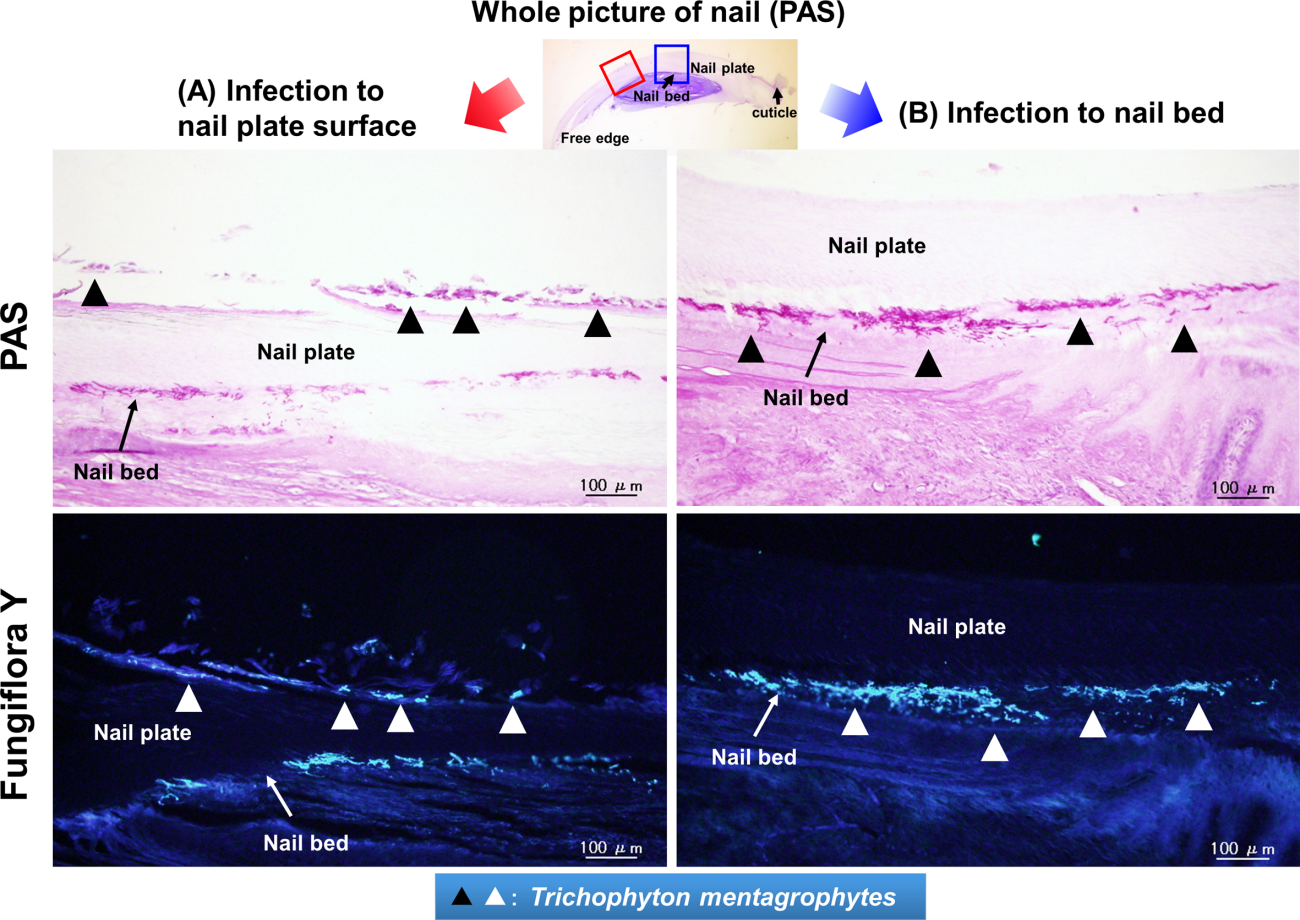
Fungi detection by light and fluorescence microscopy in the nails of a *Trichophyton mentagrophytes*-infected guinea pig onychomycosis model. On histopathological examination using the Periodic acid Schiff (PAS) stain and Fungiflora Y stain, fungi in the proximal nail surface (A) and the proximal nail bed (B) were found to be present in the untreated group (▲, ∆).

In the untreated group, fungal elements were observed in both nail plate surface and nail bed in the proximal regions by histopathological examination with PAS staining and Fungiflora Y staining (Fig. 3). The rate of fungal infection in all areas including the proximal nail plate was at 100%, 80%, and 60% in the subgroups with 2, 4, and 8 weeks of nontreatment period, respectively. On the other hand, the fungal infection rate in the proximal nail bed was at 33%, 40%, and 40% in the subgroups with 2, 4 and 8 weeks of non-treatment period, respectively (Table 1).

**Table 1.** The rate of fungal infection.

	Rate of the nail with infection (%)		
	Left period (week)		
Group	2	4	8
untreated	100	80	60
	Nail bed: 33	Nail bed: 40	Nail bed: 40
	Nail plate: 100	Nail plate: 80	Nail plate: 60
LLCZ	0^**^	0^*^	50
	Nail bed: 0	Nail bed: 0	Nail bed: 0
	Nail plate: 0	Nail plate: 0	Nail plate: 50
EFCZ	0^**^	33	60
	Nail bed: 0	Nail bed: 33	Nail bed: 20
	Nail plate: 0	Nail plate: 33	Nail plate: 60

The values in this table show the percentages of the fungi-positive nails.

Infection rates in nails were analyzed by the Fisher's extract test (one-side). *P < .05, **P < .01 versus Untreated group. N = 5–6.

LLCZ, Luliconazole 5% solution; EFCZ, Efinaconazole 10% solution.

### Histopathological characterization and infection rate of the LLCZ- and EFCZ-treated groups

**Figure 4.**
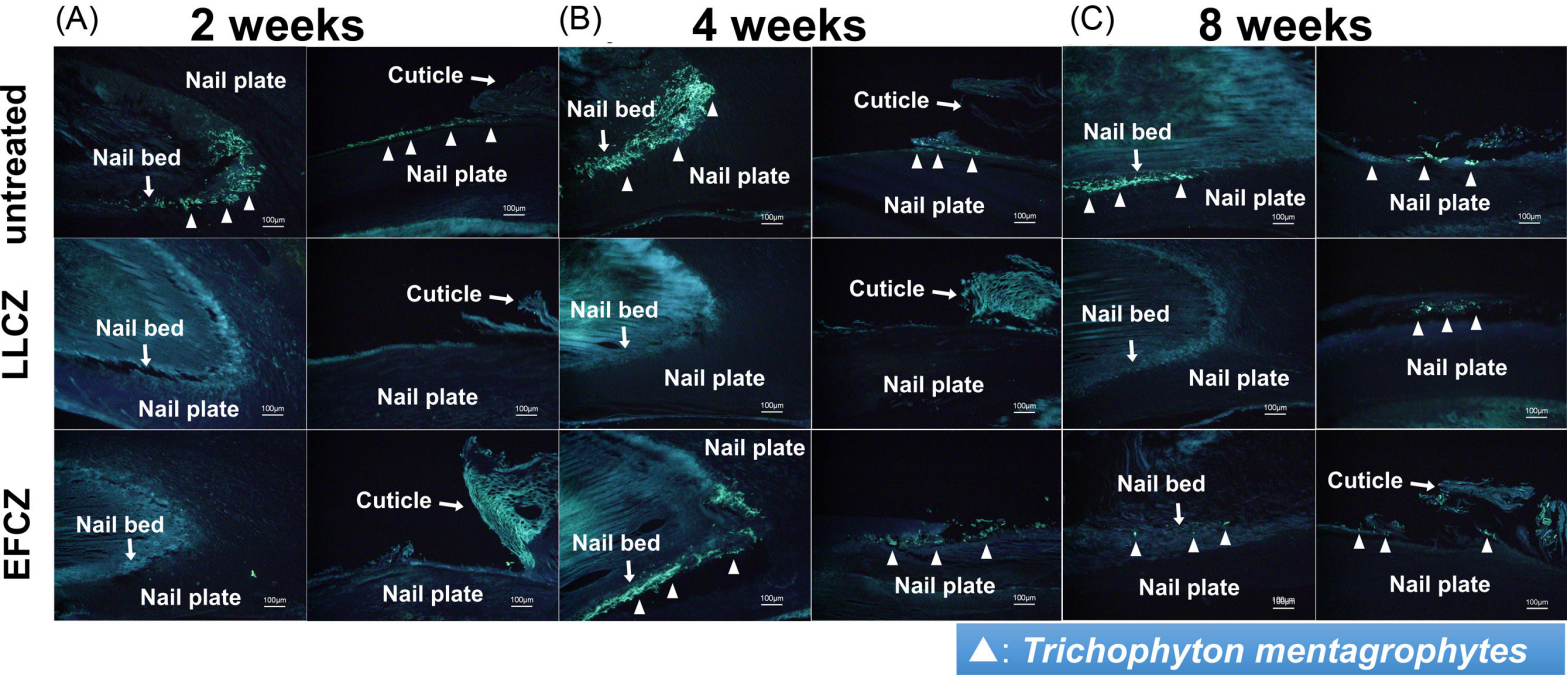
Fungi detection by fluorescence microscopy in the nails using histopathological examination by Fungiflora Y stain. The subgroups with 2 weeks (A), 4 weeks (B), and 8 weeks (C) of the nontreatment period after dosing with LLCZ and EFCZ. Fungi in the proximal nail bed (A) and the proximal nail surface (B) were found (∆). LLCZ, Luliconazole 5% solution; EFCZ, Efinaconazole 10% solution.

No fungal element was found in the subgroups with 2 weeks of the nontreatment period following either drug treatment (Fig. 4A, Table 1). In the subgroups with 4 weeks of the nontreatment period, fungi in the nails were not seen in the subgroup treated with LLCZ, whereas some nails having fungal infection were found in the subgroup treated with EFCZ. The infection rate in the EFCZ treated subgroup was 33% (Table 1, Fig. 4B).

In the subgroups with 8 weeks of the nontreatment period, evaluated nails showed different fungal distributions in the tissues between the drug groups. No fungus was found in the nail bed but 50% infection rate was detected in the nail plate in the LLCZ-treated group. The fungal infection rate in the EFCZ treated group was 20% in the nail bed and 60% in the nail plate (Fig. 4C and Table 1).

## Discussion

There are a few papers that are related to onychomycosis treatment using animal models,^15^^,^^17–21^ however, none of them have evaluated the lasting preventive effects of topical antifungal drugs. In addition, no guinea pig models have demonstrated the pathological pattern found in many patients diagnosed with different types of onychomycosis. In this experiment, histopathological examination showed that the pathological pattern of the infected nail in this animal model was similar to those in the rare clinical disease patterns, such as PSO and SWO rather than distal and lateral subungual onychomycosis (DLSO).

This animal model was established by the inoculation of *T. mentagrophytes* TIMM2789 that is known as zoophilic race,^28^ It is generally known that anthropophilic fungi, *T. interdigitale* and *T. rubrum*, are the main causes of human onychomycosis.^29^ Although there may be some differences between these fungi, the observed morphological changes in the nails of the guinea pigs were quite similar to those in human nails. We found histopathologically that the inoculated fungi around the whole nail infected into deeper regions in the nail tissue. This characteristic infection at deeper regions was made by fungi entered from the side such as lateral nail fold, but not from superficial nail layer to deep nail. It is obvious that fungi at the inner of nail plate were not found in all nails containing untreated group histopathologically. These findings demonstrate that this established animal model shares some characteristics with those in human onychomycosis as a pre-clinical model, and that the results in this study of LLCZ and EFCZ could be extrapolated to human clinical evaluation of these antifungal drugs.

LLCZ showed longer-lasting preventive effects on fungal infection than EFCZ, especially at the deeper region of the nail bed. This phenomenon contradicts the known pharmacokinetics of LLCZ applied to the nail surface. The applied LLCZ would be difficult to reach the deeper region as it is trapped in the nail tissue due to its strong keratin affinity.^11^ It is known that EFCZ, of which keratin affinity is low, is easier to permeate into the deeper region of the nail without being trapped.^11^^,^^15^

The contradiction between the kinetics and pharmaceutical efficacy in these drugs could be explained by the following pharmacokinetic hypothesis in LLCZ. Luliconazole can be retained in the nail as a compound reservoir by its keratin affinity, and would be continuously desorbed and gradually delivered to the deeper site of nail bed to exhibit its antifungal effects.^16^ This gradual compound release could contribute to the longer-lasting efficacy in LLCZ. In contrast, EFCZ would be easier to leave from the infection site due to its lower keratin affinity.^11^^,^^15^ These kinetic differences would create different effective compound concentrations in the tissue site in the subgroups with 4 and 8 weeks of non-treatment period after the drug treatment.

Pharmacological characteristics would also contribute to the preventive effects in the subgroups with 8 weeks of non-treatment period. Luliconazole has a very high antifungal activity in the range of 0.00049 to 0.002 µg/mL as minimum inhibitory concentration (MIC) *in vitro* against *T. mentagrophytes*.^13^ The MIC of the strain (TIMM2789) used in the model of this study was 0.00098 µg/mL. On the other hand, the inhibitory concentration for efinaconazole is 0.0039 to 0.031 µg/mL *in vitro* against *T. mentagrophytes*.^13^ The MIC of the strain (TIMM2789) used in the model of this study was 0.031 µg/mL. This higher activity in luliconazole would be possible to achieve the curative efficacy of onychomycosis even at its lower concentration as it remained in the nail tissue longer as described above. The MIC value of efinaconazole is higher than that of luliconazole; thus, the concentration of efinaconazole in the nail tissue will fall under its MIC level, reducing efficacy in the subgroups with the longer nontreatment period of 4 or 8 weeks.

In conclusion, LLCZ prevented the fungal infection longer than EFCZ did in the *T. mentagrophytes*-infected guinea pig onychomycosis model. These results could support the prophylactic concept clinically to prevent the recurrence by topical antifungal drugs.^5^^,^^8–12^

## References

[1] Ghannoum MA , Hajjeh RA, Scher R et al. A large-scale North American study of fungal isolates from nails: the frequency of onychomycosis, fungal distribution, and antifungal susceptibility patterns. J Am Acad Dermatol. 2000; 43: 641–648. 11004620 10.1067/mjd.2000.107754

[2] Papini M , Piraccini BM, Difonzo E et al. Epidemiology of onychomycosis in Italy: prevalence data and risk factor identification. Mycoses. 2015; 58: 659–664. 26412300 10.1111/myc.12396

[3] Maraki S , Mavromanolaki VE. Epidemiology of onychomycosis in Crete, Greece: a 12-year study. Mycoses. 2016; 59: 798–802. 27432461 10.1111/myc.12533

[4] Watanabe S , Harada T, Hiruma M et al. Epidemiological survey of foot diseases in Japan: Results of 30000 foot checks by dermatologists. J Dermatol. 2010; 37: 397–406. 20536644 10.1111/j.1346-8138.2009.00741.x

[5] Piraccini BM , Sisti A, Tosti A. Long-term follow-up of toenail onychomycosis caused by dermatophytes after successful treatment with systemic antifungal agents. J Am Acad Dermatol. 2010; 62: 411–414. 20159308 10.1016/j.jaad.2009.04.062

[6] Scher RK , Baran R. Onychomycosis in clinical practice: factors contributing to recurrence. Br J Dermatol. 2003; 149(suppl65): 5–9. 10.1046/j.1365-2133.149.s65.5.x 14510969

[7] Gupta AK , Simpson FC. New therapeutic options for onychomycosis. Expert Opin Pharmacother. 2012; 13: 1131–1142. 22533461 10.1517/14656566.2012.681779

[8] Shemer A , Gupta AK, Kamshov A et al. Topical antifungal treatment prevents recurrence of toenail onychomycosis following cure. Dermatol Ther. 2017; 30. 10.1111/dth.12545 28856784

[9] Arroll B , Oakley A. Preventing long term relapsing tinea unguium with topical antifungal cream: a case report. Cases J. 2009; 2: 70. 19154619 10.1186/1757-1626-2-70 PMC2647914

[10] Sigurgeirsson B , Olafsson J, Steinsson J et al. Efficacy of amorrolfine nail lacquer for the prophylaxis of onychomycosis over 3 years. J Eur Acad Dermatol Venereol. 2010; 24: 910–915. 20028447 10.1111/j.1468-3083.2009.03547.x

[11] Matsuda Y , Sugiura K, Hashimoto T et al. Efficacy coefficients determined using nail permeability and antifungal activity in keratin-containing media are useful for predicting clinical efficacies of topical drugs for onychomycosis. Pros one. 2016; 11. 10.1371/journal.pone.0159661 PMC4956321 27441843

[12] Tosti A , Elewski BE. Onychomycosis: Practical approaches to minimize relapse and recurrence. Skin Appendage Disord. 2016; 2: 83–87. 27843933 10.1159/000448056 PMC5096127

[13] Maeda J , Nanjoh Y, Koga H et al. In vitro antifungal activity of luliconazole against Trichophyton spp: comparative study using a combination test method to determine the MIC and MFC of topical antifungal drugs. Med Mycol J. 2016; 57: J1–J6. 26936346 10.3314/mmj.57.J1

[14] Koga H , Nanjoh Y, Makimura K et al. In vitro antifungal activities of luliconazole, a new topical imidazole. Med Mycol. 2009; 47: 640–647. 19115136 10.1080/13693780802541518

[15] Sugiura K , Sugimoto N, Hosaka S et al. The low keratin affinity of efinaconazole contributes to its nail penetration and fungicidal activity in topical onychomycosis treatment. Antimicrob Agents Chemother. 2014; 58: 3837–3842. 24752277 10.1128/AAC.00111-14 PMC4068573

[16] Hasuko M , Shiomi R, Takahashi Y et al. Affinity of luliconazole for human nail derived keratin. Med Mycol J. 2017; 58J: J113–J119. 10.3314/mmj.17-00009 29187718

[17] Tatsumi Y , Yokoo M, Senda H et al. Therapeutic efficacy of topically applied KP-103 against experimental tinea unguium in guinea pigs in comparison with amorolfine and terbinafine. Antimicrob Agents Chemother. 2002; 46: 3797–3801. 12435679 10.1128/AAC.46.12.3797-3801.2002 PMC132781

[18] Shimamura T , Kubota N, Nagasaka S et al. Establishment of a novel model of onychomycosis in rabbits for evaluation of antifungal agents. Antimicrob Agents Chemother. 2011; 55: 3150–3155. 21555762 10.1128/AAC.00399-11 PMC3122464

[19] Shimamura T , Hasegawa N, Kubota N. Antifungal activity of luliconazole nail solution on in vitro and in vivo onychomycosis model. Med Mycol J. 2016; 57J: J13–J18. 10.3314/mmj.57.J13 26936347

[20] Tachibana H , Kumagai N, Tatsumi Y. Fungicidal activity in the presence of keratin as an important factor contributing to in vivo efficacy: A comparison of efinaconazole, tavaborole, and ciclopirox. J Fungi. 2017; 58. 10.3390/jof3040058 PMC5753160 29371574

[21] Lee BC , Pangeni R, Na J et al. Preparation and in vivo evaluation of a highly skin- and nail-permeable efinaconazole topical formulation for enhanced treatment of onychomycosis. Drug Deliv. 2019; 26: 1167–1177. 31738083 10.1080/10717544.2019.1687612 PMC6882438

[22] Iwanaga T , Ushigami T, Anzawa K et al. Pathogenic dermatophytes survive in nail lesions during oral terbinafine treatment for tinea unguium. Mycopathologia. 2017; 182: 673–679. 28281037 10.1007/s11046-017-0118-8 PMC5500682

[23] Iwanaga T , Ushigami T, Anzawa K et al. Viability of pathogenic dermatophytes during a 4-week treatment with 1% topical luliconazole for tinea pedis. Med Mycol. 2019; 0: 1–3. 10.1093/mmy/myz056 PMC7108760 31111903

[24] Yoshimura R , Ito Y, Morishita N et al. Comparative study between culture and PCR-RFLP Analysis on Identification of the causative agent of tinea unguium. Med Mycol J. 2006; 47J: J1G. 10.3314/jjmm.47.11 16465135

[25] Iwanaga T , Anzawa K, Mochizuki T. Quantification of dermatophyte viability for evaluation of Antifungal Effect by Quantitative PCR. Mycopathologia. 2014; 177: 241–249. 24760383 10.1007/s11046-014-9745-5

[26] Kawamoto T. Use of a new adhesive film for the preparation of multi-purpose fresh-frozen sections from hard tissues, whole-animals, insects and plants. Arch Histol Cytol. 2003; 66: 123–143. 12846553 10.1679/aohc.66.123

[27] Okamoto M , Kamoi M, Yamachika S et al. Efficacy of Fungiflora Y staining for the diagnosis of oral erythematous candidiasis. Gerodontology. 2013; 30: 220–225. 22582792 10.1111/j.1741-2358.2012.00668.x

[28] Fujita S , Matsuyama T. Experimental tinea pedis induced by non-abrasive inoculation of Trichophyton mentagrophytes arthrospores on the plantar part of a guinea pig foot. J Med Vet Mycol. 1987; 25: 203–213. 3668758 10.1080/02681218780000541

[29] Sei Y . 2006 Epidemiological survey of dermatomycoses in Japan. Med Mycol J. 2012; 53J: J185–J192. 10.3314/mmj.53.185 23149353

